# A Novel Parameter-Variabled and Coupled Chaotic System and Its Application in Image Encryption with Plaintext-Related Key Concealment

**DOI:** 10.3390/e26100832

**Published:** 2024-09-30

**Authors:** Zuxi Wang, Siyang Wang, Zhong Chen, Boyun Zhou

**Affiliations:** 1 National Key Laboratory of Multispectral Information Intelligent Processing Technology, Wuhan 430074, China; zuxiw@mail.hust.edu.cn (Z.W.); m202273164@hust.edu.cn (S.W.); 2 Key Laboratory of Image Information Processing and Intelligent Control, Ministry of Education of China, Wuhan 430074, China; 3 School of Artificial Intelligence and Automation, Huazhong University of Science and Technology, Wuhan 430074, China; 4 School of Engineering, University of Glasgow, Glasgow G12 8QQ, UK; zhouby0505@163.com

**Keywords:** chaotic system, parameter-variabled coupled chaotic system, chaos-based pseudo-random sequence generator, image encryption, key reusability

## Abstract

The design of a chaotic system and pseudo-random sequence generation method with excellent performance and its application in image encryption have always been attractive and challenging research fields. In this paper, a new model of parameter-variabled coupled chaotic system (PVCCS) is established by interaction coupling between parameters and states of multiple low-dimensional chaotic systems, and a new way to construct more complex hyperchaotic systems from simple low-dimensional systems is obtained. At the same time, based on this model and dynamical DNA codings and operations, a new pseudo-random sequence generation method (PSGM-3DPVCCS/DNA) is proposed, and it is verified that the generated pseudo-random sequence of PSGM-3DPVCCS/DNA has excellent random characteristics. Furthermore, this paper designs a novel pixel chain diffusion image encryption algorithm based on the proposed parameter-variabled coupled chaotic system (PVCCS) in which the hash value of plaintext image is associated with the initial key to participate in the encryption process so that the encryption key is closely associated with plaintext, which improves the security of the algorithm and effectively resists the differential cryptanalysis risk. In addition, an information hiding method is designed to hide the hash value of plaintext image in ciphertext image so that the hash value does not need to be transmitted in each encryption, and the initial key can be reused, which solves the key management problem in application and improves the application efficiency of the encryption algorithm. The experimental analysis shows that the chaotic system constructed in this paper is creative and universal and has more excellent chaotic characteristics than the original low-dimensional system. The sequence generated by the pseudo-random sequence generation method has excellent pseudo-random characteristics and security, and the image encryption algorithm can effectively resist differential cryptanalysis risk, showing advanced encryption performance.

## 1. Introduction

Chaotic systems have been widely employed in image encryption due to their attributes of uncertainty, unpredictability, and irreducibility. In 1998, Fridrich et al. [1] pioneered the use of chaotic systems to generate random sequences for encrypted images, opening a new path for image encryption. Subsequently, various chaotic systems, including logistic chaotic mapping, Henon chaotic mapping, Lorenz chaotic mapping, and other chaotic mappings, have continuously been applied in the field of image encryption such as [2,3,4].

While many low-dimensional chaotic systems are characterized by a limited key space and simpler spatial structures, which can make them susceptible to exhaustive attacks, this does not mean that all low-dimensional systems are inherently weak. Recent advancements have shown that some low-dimensional chaotic systems can achieve robust chaos, providing sustained and unpredictable behavior that enhances their security. Several studies [5,6,7] have proposed different methods for generating random sequences based on multiple one-dimensional or two-dimensional systems, coupled mappings, and so on. For instance, a two-dimensional Baker mapping was proposed in [5] to generate random sequences, along with examples of other two-dimensional chaotic mappings, providing insights for designing multidimensional chaotic mappings. The use of two-dimensional sinusoidal logistic chaos mappings was proposed in [6], addressing the drawbacks of poor randomness and relatively simple trajectories observed in one-dimensional logistic chaos mappings. The authors of [7] proposed a new one-dimensional chaotic system called the One-Dimensional Zero Exclusion Chaotic Mapping (1D-ZECM), which has the characteristics of approximate global chaos, wide chaos range, and high Lyapunov exponent. However, despite the excellent performance of these low-dimensional chaotic systems, there are still some drawbacks, such as the small key space of low-dimensional chaotic systems. Therefore, the coupling method proposed in this paper can significantly improve the spatial complexity and key space of low-dimensional chaotic systems (such as logistic).

Conversely, high-dimensional mappings such as Henon chaotic mappings, Lorenz chaotic mappings, and certain hyperchaotic systems offer complex and unpredictable trajectories, which contribute to their robustness. In [8], a combination of composite chaos and hyperchaos was proposed for image encryption. The authors of [9] suggested coupling chaotic systems with cosine transforms to enhance the chaotic characteristics, while [10] introduced a combination of chaotic systems and AES algorithms to increase the key space and chaotically distribute the encryption block and key locations, thus improving the security level. In [11,12], hyperchaotic mappings were employed to generate random sequences with larger key spaces, exhibiting complex trajectories and good chaotic properties. Although the security of the schemes proposed in these papers is high, their structures are relatively complex and challenging to implement in hardware. In this paper, we present a parameter-variabled coupled chaotic system, which has the simplest structure among logistic chaotic systems while maintaining a relatively simple structure without sacrificing security. However, the implementation of these high-dimensional systems can be prohibitively expensive due to their significant hardware requirements. Thus, while high-dimensional chaotic systems can offer superior security features, practical considerations often limit their applicability.

Excellent random sequence generation algorithms form the foundation of image encryption algorithms. However, outstanding image encryption algorithms also rely on a more intricate and random encryption process. The authors of [13] proposed the application of DNA coding operations in the field of image encryption, which offers characteristics such as high storage density, high parallelism, and a strong anti-attack effect. Researchers have proposed encryption algorithms that combine various chaotic systems and DNA coding operations, achieving robust encryption and showing promising applications in the literature [14,15,16,17,18,19,20,21,22,23,24]. Nevertheless, the lack of connection between the key and the plaintext makes these algorithms susceptible to breaking through differential attacks. The authors of [25] introduced a differential attack method for [14,15,16,17,18,19,20,21,22,23,24], exploiting the drawback of the absence of a connection between plaintext and key in these studies. This method can be used to obtain the equivalent encryption matrix by encrypting the plaintext image with specific distinctions, thereby compromising the security of the algorithm.

To resist differential attacks and strengthen the connection between the plaintext image and the encryption process, [26,27,28,29,30] and others have proposed using hash functions such as SHA-256 or MD5. They utilize the hash of the plaintext image to generate the initial value of the chaotic system, significantly strengthening the connection between the plaintext and the key. Any slight change in the plaintext produces a very different ciphertext image, demonstrating extreme resistance to differentials. However, some problems in these papers need to be addressed.

The encryption schemes in these papers require regenerating the key each time the plaintext image is encrypted. The encryptor must repeatedly pass the key to the decryptor, making these schemes essentially a one-at-a-time image encryption algorithm. It is well known that the key distribution problem of the one-at-a-time algorithm is complicated, and many critical transfers are extremely inconvenient in practical applications. Hence, it is necessary to study symmetric image encryption algorithms whose keys can be reused.

Based on the aforementioned scenario, this paper presents an image encryption algorithm that utilizes a parameter-variabled logistic chaotic mapping model and DNA coding. The algorithm involves generating bifurcation coefficients of the chaotic mapping by employing the SHA-256 hash function. These coefficients are combined with the initial key value to produce a pseudo-random sequence. The obtained random sequence is then used to displace the plaintext image, followed by applying DNA coding for diffusion operations on the resulting ciphertext image. Finally, the diffused ciphertext image and the plaintext image are merged in chunks using the Chinese remainder theorem [31,32] to obtain the final ciphertext image.

The main contributions of this paper are as follows:

- A new parameter-variabled coupled chaotic system (PVCCS) is proposed, which involves interaction coupling between parameters and states of multiple low-dimensional chaotic systems, thus obtaining a higher-dimensional chaotic system. Moreover, this coupling structure is extensible and can be extended to the state coupling of any different basic chaotic systems.
- A pseudo-random sequence generation method (PSGM-3DPVCCS/DNA) is proposed based on the proposed chaotic system (3DPVCCS) and dynamical DNA codings and operations. Compared with the traditional pseudo-random sequence generation method, our proposed method shows various characteristics very similar to real random sequences.
- A new pixel-linked diffusion image encryption algorithm is designed. In the algorithm, encryption and decryption operations are designed based on a parameter-variabled coupled chaotic system (3DPVCCS) and dynamical DNA coding and operation. The security analysis proves that the proposed algorithm shows exceptionally high security.
- Simultaneously, the proposed encryption algorithm leverages the hash value of the plaintext image to generate subkeys, thereby enhancing the system’s resistance to differential cryptanalysis but also, for the first time, addresses the key distribution issue that is overlooked in other studies by concealing it within the ciphertext image.

## 2. PVCCS

This section introduces a general model of a parameter-variabled coupled chaotic system (PVCCS). A concrete three-dimensional parameter-variabled coupled chaotic system based on a one-dimensional logistic function (3DPVCCS) and analysis are also given.

### 2.1. A General Model of Parameter-Variabled Coupled Chaotic System (PVCCS)

PVCCS is presented to obtain a chaotic system with a more complex chaotic behavior, which is based on several simple low-dimensional chaotic systems.

We suppose there are *n* one-dimemsional simple chaotic systems:

(1) $$x_{k}\left(i+1\right)=f_{k}\left(x_{k}(i);\mu_{k}\left(i\right)\right),\;k=1,2,\ldots ,n.$$

where, without loss of generality, we suppose that all functions $f_{k}$ are surjections from (0,1) to (0,1), and the value ranges of all parameters $\mu_{k}$ are the same as [a,b].

Based on the n one-dimensional simple chaotic systems (1), a general parameter-variabled coupled chaotic system is constructed as follows:

(2) $$\left\{\begin{array}{c} x_{1}\left(i+1\right)=f_{1}\left(y_{1}(i);\mu_{1}\left(i\right)\right)\\ x_{2}\left(i+1\right)=f_{2}\left(y_{2}(i);\mu_{2}\left(i\right)\right)\\ \vdots \\ x_{n}\left(i+1\right)=f_{n}\left(y_{n}(i);\mu_{n}\left(i\right)\right) \end{array}\right.$$

where $y_{k}\left(i\right)$ is the state variable coupled by states $x_{1}\left(i\right)$,…,$x_{n}\left(i\right)$. $\mu_{k}\left(i\right)$ is the variabled parameter of the system determined by states $x_{1}\left(i\right)$,…,$x_{n}\left(i\right)$. A more detailed description of (2) is as follows.

System (2) describes a general coupled system. We describe System (2) and the coupling manner in vector form as follows:

(3) $$X\left(i+1\right)≜\left(\begin{array}{c} x_{1}\left(i+1\right)\\ x_{2}\left(i+1\right)\\ \vdots \\ x_{n}\left(i+1\right) \end{array}\right)=F\left(Y(i);\mu (i)\right)≜\left(\begin{array}{c} f_{1}\left(y_{1}(i);\mu_{1}\left(i\right)\right)\\ f_{2}\left(y_{2}(i);\mu_{2}\left(i\right)\right)\\ \vdots \\ f_{n}\left(y_{n}(i);\mu_{n}\left(i\right)\right) \end{array}\right).$$

where *Y*(*i*) is the vector of coupled variables, and the coupling method of states is shown as

(4) $$Y\left(i\right)≜\left(\begin{array}{c} y_{1}\left(i\right)\\ y_{2}\left(i\right)\\ \vdots \\ y_{n}\left(i\right) \end{array}\right)=W_{i}X\left(i\right),$$

where $W_{i}={\left(w_{kl}\right)}_{n\times n}$ is the state coupling matrix, $W_{i}=\delta_{i}W_{0}^{T}+\left(1-\delta_{i}\right)W_{0}$.

$\delta_{i}=\left\{\begin{array}{c} 0,i\;mod\;2=0\\ 1,i\;mod\;2=1 \end{array}\right.$, $W_{0}$ is a coupling matrix in which the sum of elements in each row and the sum of elements in each column are one. The value range of y(i) is consistent with that of x(i). In the process of the system evolution, for different *i*, the system states couple in a manner of alternating between $W_{0}$ and $W_{0}^{T}$.

And In the process of system evolution, the parameters change in the following ways:

(5) $$\mu \left(i\right)=\mu +\left(b-\mu \right)\circ \left(W_{i}^{T}X\left(i\right)\right),$$

where ∘ is the Hadamard product, $\mu ={\left(\mu_{1},\mu_{2},\ldots \mu_{n}\right)}^{T}$, $\mu_{k}\in \left[a,b\right],k=1,2,\ldots ,n$.

From the description of the system above, it can be observed that the states of System (3) are coupled in the manner described in (4), and as the system evolves, the states are alternately coupled through $W_{0}$ or $W_{0}^{T}$. Corresponding to different coupling matrices $W_{0}$, different structures of parameter-variabled coupled systems are obtained. Furthermore, from (4) and (5), it is evident that when the system states are coupled through $W_{0}$, the system parameters μ(i) are modified by coupling states through the transposed matrix $W_{0}^{T}$, resulting in a parameter-variabled system.

It is apparent that when System (3) couples its states and changes its parameters in this manner, each component of the system completely deviates from the trajectory of the original one-dimensional simple chaotic system. The states in all directions of the system are coupled and cross-influenced, which makes the state of the system more complicated and forms a high-dimensional complex chaotic system.

### 2.2. The Parameter-Variabled Coupled Chaotic System Based on the Logistic Map

For logistic map $f\left(x\right)=\mu x\left(1-x\right)$, one obtains the classical one-dimensional logistic chaotic system:

(6) $$x\left(i+1\right)=f\left(x(i);\mu \right)=\mu x\left(i\right)\left(1-x(i)\right),$$

where x(i)∈(0,1], and when μ∈(3.5699,4), Equation (6) exhibits chaotic characteristics. However, the system has a simple structure and its variations are not complex, making it difficult to resist phase space reconstruction analysis.

But if in System (3), all $f_{k}=f$ as in (6), then it leads to an n-dimensional parameter-variabled coupled chaotic system based on the logistic map, denoted as nDPVCCS, with the description as follows:

(7) $$\left\{\begin{array}{c} x_{1}\left(i+1\right)=\mu_{1}\left(i\right)y_{1}\left(i\right)\left(1-y_{1}\left(i\right)\right)\\ x_{2}\left(i+1\right)=\mu_{2}\left(i\right)y_{2}\left(i\right)\left(1-y_{2}\left(i\right)\right)\\ \vdots \\ x_{n}\left(i+1\right)=\mu_{n}\left(i\right)y_{n}\left(i\right)\left(1-y_{n}\left(i\right)\right) \end{array}\right.$$

where $y_{k}\left(i\right)$ is the state variable coupled by states $x_{1}\left(i\right)$,…, $x_{n}\left(i\right)$. $\mu_{k}\left(i\right)$ is the variabled parameter of the system determined by states $x_{1}\left(i\right)$, …, $x_{n}\left(i\right)$. And (7) can be expressed in vector form from (3)–(5) as follows:

(8) $$X\left(i+1\right)=\mu \left(i\right)\circ \left(W_{i}X\left(i\right)\right)\circ \left(1-W_{i}X\left(i\right)\right),$$

where $\mu \left(i\right)=\mu +\left(4-\mu \right)\circ \left(W_{i}^{T}X\left(i\right)\right)$,$W_{i}=\delta_{i}W_{0}^{T}+\left(1-\delta_{i}\right)W_{0}$, $\mu ={\left(\mu_{1},\mu_{2},\ldots ,\mu_{n}\right)}^{T}$, $\mu_{k}\in \left(3.5699,4\right]$, k=1,2,…,n.

In this paper, we set $W_{0}=\left(\begin{array}{ccc} 0 & 1 & 0\\ 0 & 0 & 1\\ 1 & 0 & 0 \end{array}\right)$, $X={\left(x,y,z\right)}^{T}$, $\mu ={\left(\mu_{1},\mu_{2},\mu_{3}\right)}^{T}$, obtaining a special three-dimensional parameter-variabled coupled chaotic system (3DPVCCS) with simple expressions as shown in Equation (9),

(9) $$\left\{\begin{array}{c} x_{i+1}=\left\{\begin{array}{c} \left[\mu_{1}+\left(4-\mu_{1}\right)z_{i}\right]y_{i}\left(1-y_{i}\right),i\;mod2=0\\ \left[\mu_{1}+\left(4-\mu_{1}\right)y_{i}\right]z_{i}\left(1-z_{i}\right),i\;mod2=1 \end{array}\right.\\ y_{i+1}=\left\{\begin{array}{c} \left[\mu_{2}+\left(4-\mu_{2}\right)x_{i}\right]z_{i}\left(1-z_{i}\right),i\;mod2=0\\ \left[\mu_{2}+\left(4-\mu_{2}\right)z_{i}\right]x_{i}\left(1-x_{i}\right),i\;mod2=1 \end{array}\right.\\ z_{i+1}=\left\{\begin{array}{c} \left[\mu_{3}+\left(4-\mu_{3}\right)y_{i}\right]x_{i}\left(1-x_{i}\right),i\;mod2=0\\ \left[\mu_{3}+\left(4-\mu_{3}\right)x_{i}\right]y_{i}\left(1-y_{i}\right),i\;mod2=1 \end{array}\right. \end{array}\right.$$

where $\mu_{1},\mu_{2},\mu_{3}\in \left(1,4\right]$, and at least one of $\mu_{k}\in \left(3.5699,4\right],k=1,2,3$. $x_{i},y_{i},z_{i}\in \left(0,1\right)$, i∈1,2,….

Equation (9) describes the evolution process of three one-dimensional logistic systems in which the state of one logistic system is coupled by the states of two other logistic systems and its parameters are changed by the coupling of the states of the other two logistic systems, thus forming a three-dimensional parameter-variabled coupled chaotic system (3DPVCCS).

By referring to the analysis method of chaotic properties in [33,34,35], the following part of this section analyzes and verifies that System (9) is a three-dimensional hyperchaotic system with more complex dynamic properties and better performance than the original one-dimensional logistic system based on the Lyapunov exponent, the phase diagram, the state diagram, and approximate entropy.

### 2.3. Chaos Analysis

The Lyapunov exponent (LE) is related to the average exponential divergence rate of neighboring orbits in the phase space, which provides a qualitative and quantitative characterization of the dynamical behavior. A discrete system is said to be chaotic in the sense of LE if it satisfies the two conditions [36]:

(1) its phase space region is globally bounded;

(2) it has at least one positive LE.

For 3DPVCCS System (9), states $x_{i},y_{i},z_{i}\in \left(0,1\right),i=1,2\ldots$, so the phase space region of (9) is globally bounded, which satisfies Condition (1) of the definition of chaotic in the sense of LE above.

For verifying Condition (2), we calculate and analyze the Lyapunov exponent (LE) in three component directions of System (9).

The LE is given by

(10) $$LE=\lim_{n\to \infty }\frac{1}{n}\sum_{i=1}^{n}\ln\left(\frac{\Delta x_{i}}{\Delta x_{0}}\right)$$

This represents the Lyapunov exponent (LE), where $\Delta x_{i}$ is the separation between trajectories at Step i and $\Delta x_{0}$ is the initial separation.

**Figure 1:**
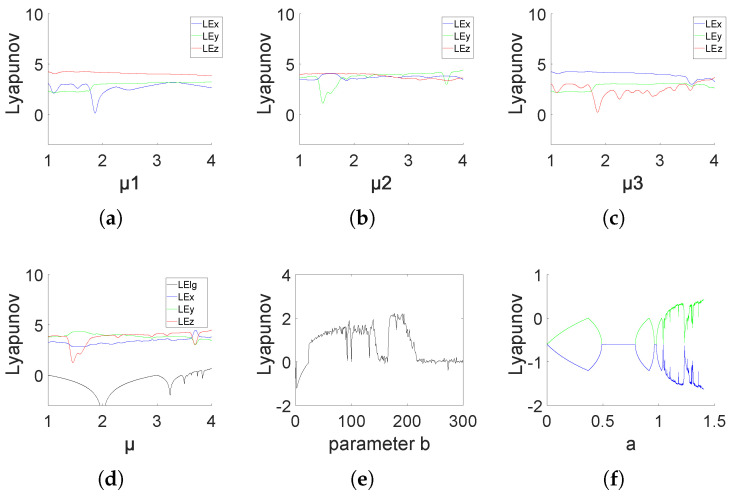
The curve of the Lyapunov exponent when the initial values (x, y, z) are set to (0.11, 0.22, 0.33), and parameter $\mu_{i}$ varies and other two parameters fixed. The fixed values of parametes $\mu_{1}$, $\mu_{2}$, $\mu_{3}$ are set as 3.982, 2.871, 3.793 respectively. In the figure caption, “LEx” represents the Lyapunov exponent on the x-axis of the 3DPVCCS, “LEy” represents the Lyapunov exponent on the y-axis of the 3DPVCCS, “LEz” represents the Lyapunov exponent on the z-axis of the 3DPVCCS, and “LElg” represents the Lyapunov exponent of the logistic system. (**a**–**c**) depict the Lyapunov exponent plots of 3DPVCCS as one of its parameters varies. (**d**) presents a comparison of the Lyapunov exponents between logistic and 3DPVCCS. (**e**) Lyapunov exponents of Lorenz, (**f**) Lyapunov exponents of Henon (The green line in the figure represents the Lyapunov exponent of the first dimension of the Henon mapping, while the blue line represents the Lyapunov exponent of the first dimension of the Henon mapping).

Figure 1 presents the Lyapunov exponents of (9) and the original logistic system. From Figure 1 (a)–(d), it can be seen that, although (9) is a system coupled by three one-dimensional logistic chaotic systems, the Lyapunov exponents (LEs) in three component directions of (9) are all positive for all parameters in [1, 4], and the three LEs are all much higher than that of the original one-dimensional logistic system. This indicates that the newly generated chaotic mapping exhibits hyperchaotic behavior, and 3DPVCCS breaks the characteristic that logistic chaos only exhibits chaotic properties at μ∈(3.5699,4]. Therefore, the coupling method presented in this paper can couple low-dimensional chaotic systems into high-dimensional chaotic systems and has better chaotic characteristics.

In addition, through the Lyapunov exponents of the Lorzen and Henon systems shown in Figure 1e,f, we can also draw a conclusion that by coupling a simple logistic system, we can make the chaotic performance of the coupled system exceed that of classical systems such as Lorzen and Henon.

### 2.4. Phase Diagram and Chaotic State Diagram

**Figure 2:**
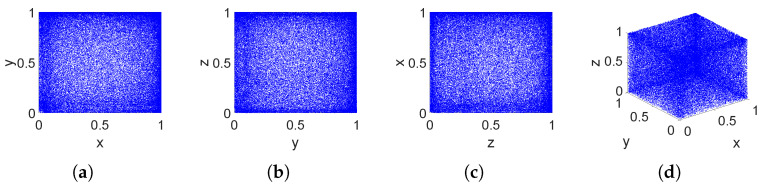
Phase diagrams of system 3DPVCCS when $\mu_{1}=3.982,\mu_{2}=2.871,\mu_{3}=3.793$ and initial values ($x_{0},y_{0},z_{0}$) are set as 0.1, 0.3, 0.5. (**a**) Chaotic phase diagram in the X-Y direction. (**b**) Chaotic phase diagram in the Y-Z plane. (**c**) Chaotic phase diagram in the Z-X plane. (**d**) 3D chaotic phase plane.

**Figure 3:**
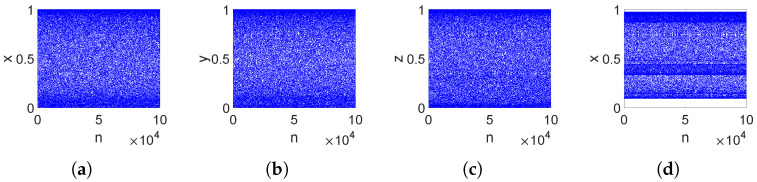
The comparison of the chaotic state diagrams of two systems with the same initial value. (**a**–**c**) State diagram on three directions of 3DPVCCS at $\mu_{1}=3.982,\mu_{2}=3.271,\mu_{3}=3.793$. (**d**) State diagram of Logistic chaos system at μ=3.91.

Phase diagrams and state diagrams can directly describe the dynamic behavior of chaotic systems. The phase diagram of a chaotic system is its non-closed trajectory in a two-dimensional phase plane or a three-dimensional phase space, and it is shown in Figure 2. The state diagram reflects the complexity of the chaotic system in space, and it is shown in Figure 3.

It can be observed that the attractor of the 3DPVCCS fills the entire phase space and distributes uniformly. Moreover, the chaotic state diagram also exhibits the characteristics of a uniform distribution of species in space. This implies that the method of parameter-variabled logistic chaotic coupling mapping proposed in this paper disrupts the original phase space of the mapping. Figure 2 contains all noise-like patterns, which means that the chaotic behavior of this variable structure system is sufficiently complex.

### 2.5. Bifurcation Diagram

**Figure 4:**
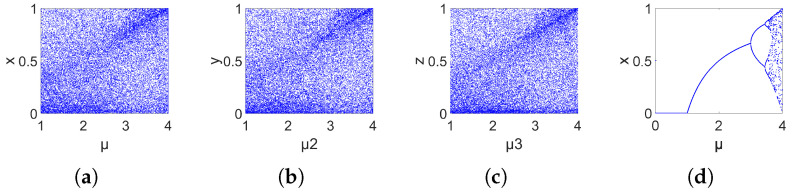
(**a**–**c**) The bifurcation diagrams of 3DPVCCS when the initial values (x, y, z) are set to (0.11, 0.22, 0.33), and parameter $\mu_{i}$ varies and other parameters are fixed. The fixed values of parametes $\mu_{1}$, $\mu_{2}$, $\mu_{3}$ are set as 3.982, 2.871, 3.793, respectively. (**d**) The bifurcation diagrams of Logistic varies with the change of μ when the initial value of *x* is set to 0.5.

By observing the bifurcation diagram, we can clearly obtain the chaotic characteristics of the system under different parameters. The more complex the bifurcation diagram, the stronger the chaotic characteristics of the system, as shown in Figure 4. It can be seen from the figure that 3DPVCCS exhibits chaotic behavior under all parameters within the value range.

### 2.6. Approximate Entropy

Approximate entropy (ApEn) is a nonlinear dynamic indicator used to quantify the regularity and unpredictability of time series fluctuations. The more complex the time series, the greater the approximate entropy. The calculation formula for approximate entropy (ApEn) is as follows:

1. **Similarity Calculation**:

(11) $$\Phi^{m}\left(r\right)=\frac{1}{N-m+1}\sum_{i=1}^{N-m+1}\ln\left(C_{i}^{m}\left(r\right)\right)$$

where $C_{i}^{m}\left(r\right)$ represents the proportion of vectors similar to the *i*th vector, *N* is the length of the time series, *m* is the embedding dimension, and *r* is the distance threshold.

2. **Approximate Entropy Calculation**:

(12) $$\mathrm{ApEn}\left(m,r\right)=\Phi^{m}\left(r\right)-\Phi^{m+1}\left(r\right)$$

**Figure 5:**
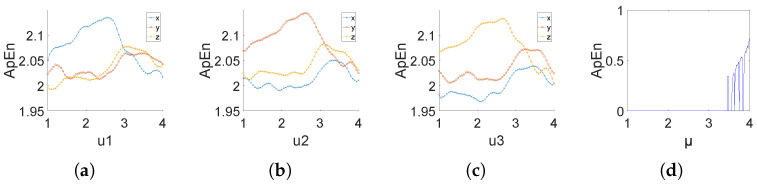
(**a**–**c**) The approximate entropy of 3DPVCCS when the initial values (x, y, z) are set to (0.11, 0.22, 0.33), and parameter $\mu_{i}$ varies and other parameters are fixed. The fixed values of parametes $\mu_{1}$, $\mu_{2}$, $\mu_{3}$ are set as 3.982, 2.871, 3.793, respectively. (**d**) The approximate entropy of the logistic varies with the change of μ when the initial value of *x* is set to 0.5.

In this article, when the initial value is set to (0.11, 0.22, 0.33), the approximate entropy results are shown in Figure 5. As can be seen from the figure, 3DPVCCS has high ApEn values in all parameter ranges. The conclusion is that the novel 3DPVCCS map can generate sequences with high complexity over a wide range of parameters.

## 3. The Method of Generating Pseudo-Random Sequences (PSGM-3DPVCCS/DNA)

### 3.1. PSGM-3DPVCCS/DNA

In biology, a DNA molecule consists of four bases: adenine (A), thymine (T), guanine (G), and cytosine (C), where A is complementary to T and C is complementary to G. Therefore, the DNA algorithm specifies that the four bases A, C, G, and T are represented by binary 00, 01, 10, and 11, respectively. There are eight coding rules based on the principle of complementary pairing of DNA, as shown in Table 1. The DNA operation Table 2 is introduced into the generation of random sequences and image encryption, enhancing the randomness of pseudo-random sequences and the complexity of image encryption.

The proposed pseudo-random sequence generation method, based on a coupled chaotic system with parameter-variabled and DNA encoding, generates chaotic sequences using PVCCS and logistic chaotic system, respectively. The sequences are then quantified separately into binary sequences. Subsequently, DNA encodes the binary sequences to obtain two DNA sequences, and a new DNA sequence is generated according to DNA operations. This resulting DNA sequence is then decrypted to obtain the final output 0–1 sequence.

The steps for generating a pseudo-random sequence are as follows:

Step 1: To generate chaotic sequences using the 3DPVCCS, we begin the iteration by inputting the initial values $x_{0},y_{0},z_{0}$ and coefficients $\mu_{1},\mu_{2},\mu_{3}$. After removing the transient influence part, we obtain the chaotic sequence $x_{i},y_{i},z_{i}$. Next, we derive the sequence $Q_{i}$ as $x_{1},y_{1},z_{1},x_{2},y_{2},z_{2},\ldots$. Then, using the classical logistic System (6), we initiate iteration after inputting the initial value $g_{0}$ and coefficients $\mu_{4}$. After removing the transient influence part, we obtain the chaotic sequence $G_{i}$.

Step 2: Binarization of chaotic sequences: We take a sequence of length L of $Q_{i}$ and $G_{i}$ (the length of L is chosen to approximate the distribution of sequences $Q_{i}$ and $G_{i}$ for subsequences of length L), respectively, to obtain their respective median values (the median of the entire sequence approximates the empirical value) as the quantization threshold. Based on the empirical median, the chaotic sequence is quantized using Equations (13) and (14) to obtain the binarized sequences $q_{i}$ and $g_{i}$:

(13) $$q_{i}=\left\{\begin{array}{c} 0,0\leq Q_{i}\leq medium\left(Q_{i}\right)\\ 1,medium\left(Q_{i}\right)<Q_{i}\leq 1 \end{array}\right.$$

(14) $$g_{i}=\left\{\begin{array}{c} 0,0\leq G_{i}\leq medium\left(G_{i}\right)\\ 1,medium\left(G_{i}\right)<G_{i}\leq 1 \end{array}\right.$$

where medium(•) is the operation to find the median of the sequence of length L.

Step 3: The binarized sequences $q_{i}$ and $g_{i}$ are DNA-encoded, DNA-operated, and decoded every 8 bits, respectively. The final pseudo-random sequence Se is then output. The DNA encoding and operation rules are shown in Table 1 and Table 2. And the DNA encoding/decoding rule sequence number and operation rule sequence number are determined by chaotic sequence $g_{i}$.

**Figure 6:**
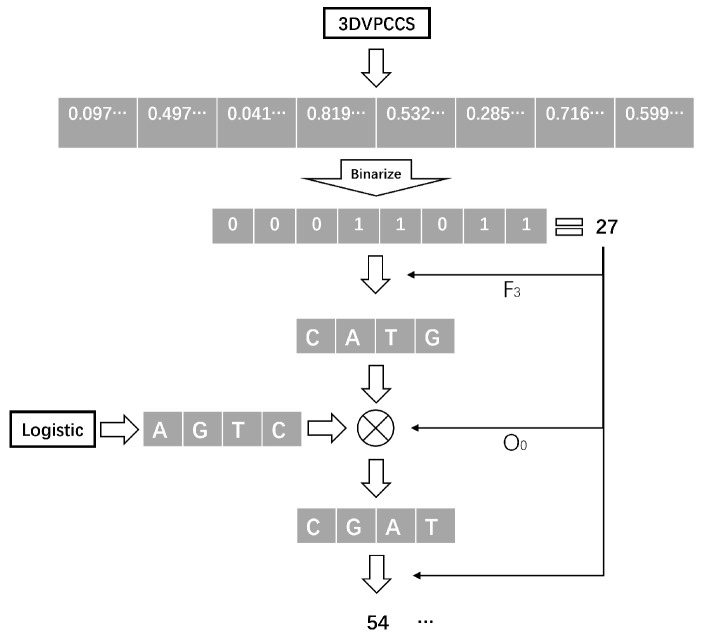
Flow chart of pseudo-random sequence generation.

An example of PSGM-3DPVCCS/DNA is shown in Figure 6.

### 3.2. Pseudo-Random Sequence Randomness Test

NIST SP800-22 [37] and TestU01 tests are utilized in this section to verify the randomness of the chaotic sequences generated by 3DPVCCS. According to the literature [38,39], NIST SP800-22 consists of a total of 15 test modules used to detect sequence randomness.

TestU01, on the other hand, is a more rigorous randomness testing suite that includes multiple sets of tests, each evaluating the randomness of the sequence from different aspects.

The sequences were tested 50 times under different initial values, with *L* = 1000, where *L* refers to the value used in Step 2 of Section 3.1. The initial values were chosen based on a random number generator for increased variability. The test results for NIST SP800-22 are presented in Table 3, and the corresponding results for TestU01 are also shown in Table 3.

In the process of implementing pseudo-random sequence generation, it is advisable to choose the value of L as large as possible, provided that implementation efficiency and application effectiveness are satisfied.

## 4. Image Encryption Based on PSGM-PVCCS/DNA and Plaintext-Related Key Concealment

This image encryption algorithm is designed for images of arbitrary size, with the plaintext image size denoted as M×N. The overall encryption scheme is described as follows.

First, the hash value of the plaintext image is combined with the first secret key to generate the initial values or parameters required for the chaotic mapping. A pseudo-random number generator based on PSGM-PVCCS/DNA is then used to generate the pseudo-random sequence needed for image encryption.

Second, based on the pseudo-random sequence and a pseudo-random selection mechanism involving multiple DNA encodings and operations, pixel scrambling and pixel diffusion operations are implemented for the image.

Lastly, utilizing the Chinese remainder theorem and the second secret key, the hash value of the plaintext image is concealed within an extended image. The final encrypted image is obtained by performing an XOR operation between the extended image and a noise image generated using the third secret key.

**Figure 7:**
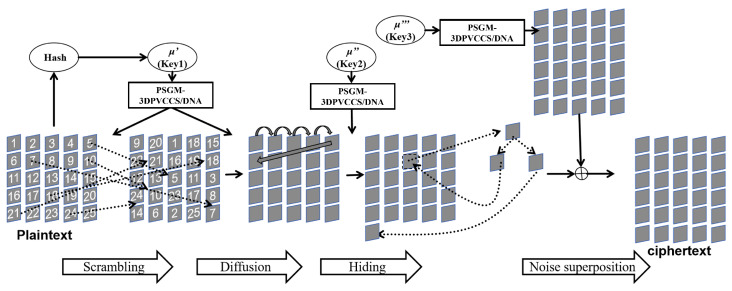
Flow chart of the image encryption algorithm.

The detailed flowchart of the image encryption process is illustrated in Figure 7, and the algorithmic steps are presented in Algorithm 1.
**Algorithm** **1** Pseudocode of image encryption based on PSGM-PVCCS/DNA and plaintext-related key Concealment**Require:** $x_{0},y_{0},z_{0},g_{0},\mu^{\prime },\mu^{\prime \prime },\mu^{\prime \prime \prime }$: Key; PT: Plaintext image; *K*: The number of rounds for shuffling and diffusion; **Ensure:** *R*: Redaction image;
  1:Produces a 0–1 sequence Se by PSGM-3DPVCCS/DNA;  2:**while** K>0 **do**  3:    Shift the sequence Se left loop by two bits to obtain the sequence Se1 and obtain the sequence *A* per 8 bits;  4:    Shuffle the image;  5:    Shift the sequence Se left loop by three bits to obtain the sequence Se2 and obtain the sequence *S* per 8 bits;  6:    Diffuse the image;  7:    Se = Se2;  8:    K=K−1;  9:**end while**10:Hide the hash in the image ${img}_{B}$;11:The image ${img}_{T}$ is masked by a noisy image ${img}_{W}$ and the result is a ciphertext image ${img}_{R}$;

### 4.1. Generating Pseudo-Random 0–1 Sequence Se Based on PSGM-3DPVCCS/DNA

We use the SHA-256 function to compress plaintext image information and generate a 256-bit sub-hash value *h*. The generated hash value *h* is divided into 16 sets of 16-bit sub-hash values, recorded as $h=(h_{1},h_{2},h_{3},\ldots ,h_{15},h_{16})$. The formula for generating coefficients $\mu_{1},\mu_{2},\mu_{3},\mu_{4}$ using parameter-variabled coupled chaotic systems is as follows:

(15) $$\left\{\begin{array}{c} \mu_{1}={\mu_{1}}^{\prime }+\frac{h_{1}⊕h_{2}⊕h_{3}⊕h_{4}}{655,360}\\ \mu_{2}={\mu_{2}}^{\prime }+\frac{h_{5}⊕h_{6}⊕h_{7}⊕h_{8}}{655,360}\\ \mu_{3}={\mu_{3}}^{\prime }+\frac{h_{9}⊕h_{10}⊕h_{11}⊕h_{12}}{655,360}\\ \mu_{4}={\mu_{4}}^{\prime }+\frac{h_{13}⊕h_{14}⊕h_{15}⊕h_{16}}{655,360} \end{array}\right.$$

where ${\mu_{i}}^{\prime }\subset \left[3,3.9\right]$, ⊕ is an XOR operator.

Then, we input the coefficients $\mu =(\mu_{1},\mu_{2},\mu_{3},\mu_{4})$ and the initial key $x_{0},y_{0},z_{0},g_{0}$ to generate a pseudo-random 01 sequence Se of length 2×M×N×c bits by PSGM-3DPVCCS/DNA, where *c* is calculated as in Equation (16):

(16) $$c=\left\lceil log_{2}\left(max\left(M,N\right)\right)\right\rceil ,$$

where the symbol ⌈⌉ indicates rounding up and max() indicates taking the larger value function.

In each subsequent cycle of the replacement and diffusion process, the sequence Se is cyclically shifted by 2 bits and selected by c-bits to obtain an integer sequence ranging from 0 to $2^{c}-1$, with a length of 2×M×N. The sequence *A* is then employed for image replacement. Simultaneously, the pseudo-random binary sequence Se is cyclically shifted by 3 bits to the left and selected by 8 bits to obtain a pseudo-random sequence *S*, ranging from 0 to 255. The sequence *S* is utilized for image diffusion.

### 4.2. Image Scrambling

Step 1: The pseudo-random sequence *A* of values between 0 and $2^{c}-1$ is generated as M×N random coordinate points $\left(X_{i},Y_{i}\right)$ according to Equation (17):

(17) $$\left\{\begin{array}{c} X_{i}=A_{2i-1}\left(\mathrm{mod}M\right)\\ Y_{i}=A_{2i}\left(\mathrm{mod}N\right) \end{array}\right.,i\in \left[1,M\times N\right].$$

Step 2: according to the following Equation (18), the plaintext image pixels scrambling operation, all the pixels in the image are exchanged with the point $\left(X_{i},Y_{i}\right)$ at the random position, resulting in the scrambled image ${img}_{D}$.

(18) $$\left\{\begin{array}{c} t=D(i,j)\\ D\left(i,j\right)=D\left(X_{(i-1)N+j},Y_{(i-1)N+j}\right)\\ D(X_{(i-1)N+j},Y_{(i-1)N+j})=t \end{array}\right.$$

where i∈[1,M],j∈[1,N].

### 4.3. Image Diffusion Based on DNA Coding Operations

We determine the DNA coding mode $F_{i}$ and the DNA operation mode $O_{J}$ in Table 1 according to Equation (19),

(19) $$\left\{\begin{array}{c} i=S(\mathrm{mod}8)\\ j=S(\mathrm{mod}3)+1 \end{array}\right.$$

The elements in the scrambled image ${img}_{D}$ and the pseudo-random 0–255 sequence S are sequentially transformed into DNA codes according to the encoding in Table 1. The post-diffusion matrix is obtained using Equation (20), which is finally decoded to obtain the diffusion matrix ${img}_{B}$,

(20) $$B_{t+1}=F_{i}^{-1}(f_{O_{j}}\left(f_{O_{j}}\left(F_{i}\left(B_{t}\right),F_{i}\left(D_{t}\right)\right),F_{i}\left(S_{t+1}\right)\right),$$

where $B_{1}=S_{1}$, t∈[1,M×N], mod is the remainder operation, $f_{O_{j}}$ is defined as a DNA operation.

### 4.4. Hash Hiding

As is known, symmetric encryption keys can be reused, but once the hash values of plaintext images participate in the encryption process, encryption keys composed of hash values of plaintext images cannot be reused. Therefore, in order to avoid this situation, this paper proposes a hash hiding scheme, which makes the initial key provided in this paper reusable. And the key used to hide the hash value comes from a part of the initial key.

Step 1: Divide the 256-bit hash value into 64 4-bit sub-hash values, each ranging in size from 0 to 15. To enhance the system’s anti-noise performance, embed each hash value eight times in a turn. A total of 512 sub-hash values are embedded into the ciphertext, denoted as $h_{i},i=1,2,\ldots ,512$.

Step 2: Utilizing PSGM-3DPVCCS/DNA, generate c-bits pseudo-random numbers $X_{i}$ and $Y_{i}$, each of length 512, with the initial key $x_{0},y_{0},z_{0},g_{0}$ and coefficients $\mu_{i}^{\prime \prime }\in \left(3.8,4\right],i=1,2,3,4$, respectively. These pseudo-random numbers are employed to obtain the image coordinates for information hiding, denoted as $\left(X_{i},Y_{i}\right)$.

(21) $$\left\{\begin{array}{c} X_{i}=X_{i}\left(\mathrm{mod}M\right)\\ Y_{i}=Y_{i}\left(\mathrm{mod}N\right) \end{array},i\in \left[1,512\right].\right.$$

Step 3: Construct congruence Equation (22) using pixel $B_{\left(X_{i},Y_{i}\right)}$ and sub-hash values $h_{i}$:

(22) $$\left\{\begin{array}{c} H_{\left(X_{i},Y_{i}\right)}=B_{\left(X_{i},Y_{i}\right)}\left(\mathrm{mod}p_{1}\right)\\ H_{\left(X_{i},Y_{i}\right)}=h_{i}\left(\mathrm{mod}p_{2}\right) \end{array}\right.$$

where $p_{1}$ and $p_{2}$ are two reciprocal positive integers and $p_{1}\geq 257$, $p_{2}\geq 17$, $p_{1}p_{2}\leq 2^{16}$.

According to the Chinese remainder theorem, a unique solution $H_{\left(X_{i},Y_{i}\right)}$ can be obtained from congruence Equation (23):

(23) $$H_{\left(X_{i},Y_{i}\right)}=\left(B_{\left(X_{i},Y_{i}\right)}{P_{1}}^{-1}P_{1}+h_{i}{P_{2}}^{-1}P_{2}\right)\mathrm{mod}P,$$

where $P=p_{1}p_{2}$, $P_{1}=\frac{P}{p_{1}}$, $P_{2}=\frac{P}{p_{2}}$, and $P_{i}^{-1}$ is the inverse of $P_{i}$ to $p_{i}$. The following Formula (24) is satisfied:

(24) $$P_{i}^{-1}P_{i}\equiv 1\left(\mathrm{mod}p_{i}\right).$$

As a result, the $h_{i}$ and $B_{\left(X_{i},Y_{i}\right)}$ information is hidden in $H_{\left(X_{i},Y_{i}\right)}$. However, because $H_{\left(X_{i},Y_{i}\right)}$ has a range of values $\left[0,p_{1}p_{2}-1\right]$, the grey value of 8-bit pixels is exceeded. To losslessly hide $H_{\left(X_{i},Y_{i}\right)}$ in pixels, perform the following:

First, use (25) to decompose $H_{\left(X_{i},Y_{i}\right)}$ into $T1_{i}$ and $T2_{i}$:

(25) $$\left\{\begin{array}{c} T1_{i}=H_{\left(X_{i},Y_{i}\right)}\left(\mathrm{mod}256\right)\\ T2_{i}=floor\left(\frac{H_{\left(X_{i},Y_{i}\right)}}{256}\right) \end{array}\right.$$

Finally, $T1_{i}$ is employed to replace the point $B_{\left(X_{i},Y_{i}\right)}$ in the original ciphertext image, while $T2_{i}$ is appended to the back of the $B_{\left(X_{i},Y_{i}\right)}$ point. The subsequent image pixels are then shifted backward one position in turn. After this process, the hidden image is synthesized, resulting in a total of 512+M×N pixels in ${img}_{T}$. If the last line is not full, it is filled with zeros.

### 4.5. Noise Image Masking

Utilizing the PSGM-3DPVCCS/DNA, a noisy image with a pixel count of $M\times (N+\left\lceil \frac{512}{M}\right\rceil )$ is generated using the initial key $x_{0},y_{0},z_{0},g_{0}$ and coefficient $\mu_{i}^{\prime \prime \prime }\in \left(3.8,4\right]$, where i=1,2,3,4. The generated noisy image is then added to the ciphertext image using Equation (26) to obtain the final ciphertext image ${img}_{R}$,

(26) $$R_{i}=W_{i}⊕T_{i}.$$

### 4.6. Image Decryption

In the process of decrypting images, it is necessary to first extract the complete hash value hidden in the ciphertext. To extract the hash value, the first step is to remove the noise mask image, which requires the participation of the key $\mu_{i}^{\prime \prime \prime },i=1,2,3,4$. Then, the hidden location $(X_{i},Y_{i}),i=1,2,\ldots M\times N$ of the hash value is determined by the key $\mu_{i}^{\prime \prime },i=1,2,3,4$, and the hash value is calculated using Equation (27). In addition, since the hash value is repeatedly embedded into the ciphertext image eight times, the extraction is also performed eight times. We determine the final hash value by selecting the case with the highest occurrence of hash values.

(27) $$\left\{\begin{array}{c} H_{\left(X_{i},Y_{i}\right)}=T1_{i}\times 256+T2_{i}\\ B_{\left(X_{i},Y_{i}\right)}=H_{\left(X_{i},Y_{i}\right)}\mathrm{mod}p_{1}\\ h_{i}=H_{\left(X_{i},Y_{i}\right)}\mathrm{mod}p_{2} \end{array},i=1,2\ldots 512\right.$$

Next, we perform inverse pixel chain diffusion. First, we use the key $\mu_{i},i=1,2,3,4$ and the hash value extracted in the previous text to calculate the true key μ. Then, we obtain the pseudo-random sequence *S*. We perform inverse diffusion according to Equation (28) to obtain the ciphertext image *D*,

(28) $$D_{t}=F_{i}^{-1}\left(f_{O_{j}}^{-1}\left(f_{O_{j}}^{-1}\left(F_{i}\left(B_{t+1}\right),F_{i}\left(B_{t}\right)\right),F_{i}\left(S_{t+1}\right)\right)\right),$$

where $B_{1}=S_{1}$, t∈[1,M×N−1], mod is the remainder operation, $f_{O_{j}}$ is defined as a DNA operation. Moreover, in the decryption stage of image diffusion based on DNA operation, the inverse operation of DNA operation $f_{O_{1}}^{-1}$ is denoted as $f_{O_{1}}$, while the inverse operation of $f_{O_{2}}^{-1}$ is $f_{O_{3}}$, and the inverse operation of $f_{O_{3}}^{-1}$ is $f_{O_{2}}$. This implies that the $f_{O_{2}}$ operation is utilized during encryption, and the $f_{O_{3}}$ operation is required for decryption and vice versa. Finally, use the key μ to obtain the pseudo-random sequence *A*. We perform inverse pixel permutation according to Equations (29) and (30) to obtain the plaintext image,

(29) $$\left\{\begin{array}{c} X_{i}=A_{2i-1}\left(\mathrm{mod}M\right)\\ Y_{i}=A_{2i}\left(\mathrm{mod}N\right) \end{array}\right.,i\in \left[1,M\times N\right].$$

(30) $$\left\{\begin{array}{c} t=D(i,j)\\ D\left(i,j\right)=D\left(X_{(i-1)N+j},Y_{(i-1)N+j}\right)\\ D(X_{(i-1)N+j},Y_{(i-1)N+j})=t \end{array}\right.$$

where i∈[1,M],j∈[1,N].

## 5. Encryption Results

This paper conducted a series of image encryption tests using the proposed algorithm on an Intel (R) Core (TM) i5-8300H CPU@2.30 GHz. The experimental software used was Windows 10 with MATLAB R2022A. The initial key values were set as [$x_{0}$, $y_{0}$, $z_{0}$, $g_{0}$] = [0.565, 0.843, 0.197, 0.421], [$\mu_{1}^{\prime }$, $\mu_{2}^{\prime }$, $\mu_{3}^{\prime }$, $\mu_{4}^{\prime }$] = [3.867, 3.493, 3.197, 4.000], [$\mu_{1}^{\prime \prime }$, $\mu_{2}^{\prime \prime }$, $\mu_{3}^{\prime \prime }$, $\mu_{4}^{\prime \prime }$] = [3.86, 3.89, 3.98, 4.0], [$\mu_{1}^{\prime \prime \prime }$, $\mu_{2}^{\prime \prime \prime }$, $\mu_{3}^{\prime \prime \prime }$, $\mu_{4}^{\prime \prime \prime }]$ = [3.97, 4.00, 3.87, 3.91]. We set the number of rounds K for shuffling and diffusion operations to three.

**Figure 8:**
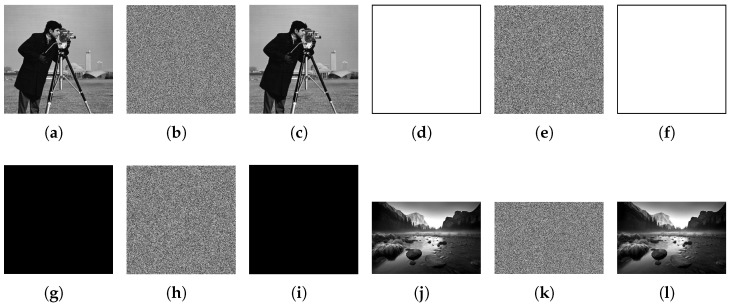
Image encryption and decryption results. (**a**) Cameraman, (**b**) Cameraman’s encryption, (**c**) Cameraman’s decryption, (**d**) White, (**e**) White’s encryption, (**f**) White’s decryption, (**g**) Black, (**h**) Black’s encryption, (**i**) Black’s decryption, (**j**) Landscape, (**k**) Landscape’s encryption, (**l**) Landscape’s decryption.

The images of Cameraman, White, Black, and Landscape were selected for encryption and decryption. The results are depicted in Figure 8. The ciphertext images produced by the encryption algorithm are complex enough to prevent leakage of plaintext information. Notably, the decrypted images precisely match the original images without distortion.

## 6. Security Analysis of Image Encryption

### 6.1. Key Space Analysis

There are 16 parameters used in the encryption scheme: chaotic initial value $x_{0}$, $y_{0}$, $z_{0}$, $g_{0}$ ∈ (0, 1), parameter $\mu_{i}^{\prime }\in \left(1,4\right],i=1,2,3,4$, bifurcation coefficients $\mu_{i}^{\prime \prime }\in \left(1,4\right],i=1,2,3,4$ and $\mu_{i}^{\prime \prime \prime }\in \left(1,4\right],i=1,2,3,4$. By setting these as the keys for this encryption algorithm and assuming that the computer maintains precision to a maximum of 15 decimal places, the size of the key space for this encryption algorithm can be estimated to be approximately $10^{237}$. This estimation is derived from the calculation $10^{15\times \left[Numberofparameters:16\right]}$. Therefore, the model is extremely resistant to exhaustive attacks. The results of the key space comparison are shown in Table 4.

### 6.2. Key Sensitivity Analysis

**Figure 9:**
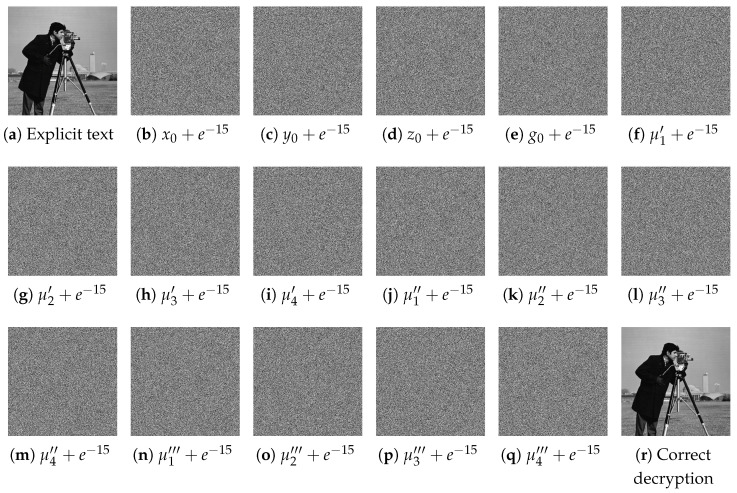
Key sensitivity analysis. (**a**) Explicit text, (**b**–**q**) are decryption images after subtle changes to a certain key, (**r**) Correct decryption.

The chosen key is used to encrypt the plaintext image, and then four experiments are carried out, respectively, each time adding a perturbation based on one of the initial values and decrypting the ciphertext image with the key containing the perturbation; the results of the four experiments are shown in Figure 9 below.

### 6.3. Analysis of the Validity of Key Hiding

In this section, we discuss the significance of key concealment in the encryption of images. Our key concealment technique allows the transmittance of the hash value of the original images alongside the encrypted images. Unlike other studies that incorporate hash values into the encryption process, such as [19,26,27,28,29,30], our method eliminates the need for a separate transmission of the hash value. This approach not only enhances security by avoiding the risk associated with additional transmissions but also simplifies the process. We integrate the hash value with the ciphertext image during transmission, which mitigates the risk of man-in-the-middle attacks and streamlines the verification process.

### 6.4. Information Entropy Analysis

Information entropy is an important parameter used to measure the randomness of a system [33], and its formula works as in Equation (31):

(31) $$H\left(m\right)=-\sum_{m_{i}=0}^{255}p\left(m_{i}\right)\log_{2}p\left(m_{i}\right),$$

where $m_{i}$ represents the pixel value and $p(m_{i})$ represents the probability that each pixel value will occur. For a 256-level grey image, the ideal entropy value should be eight, and the closer the ideal value, the more random the pixel distribution. Using this encryption algorithm, Cameraman and Black and White images are encrypted. The information entropy of the encrypted images is shown in Table 5.

Table 5 reveals that the information entropy of the ciphertext image produced by the encryption algorithm in this paper closely aligns with the theoretical value of eight. This observation underscores the uniformity of the pixel distribution generated by the designed encryption algorithm. Simultaneously, it emphasizes the minimal possibility of information leakage, showcasing the algorithm’s effectiveness in resisting security attacks.

### 6.5. Differential Attack Analysis

Differential attacks are highly effective methods for compromising encrypted images. In such attacks, subtle modifications are introduced to the original plaintext digital image data. The attacker then encrypts both the modified and original plaintext images separately using the proposed encryption algorithm. By comparing the resulting ciphertext images, the attacker aims to identify the relationship between the original plaintext image data and the encrypted ciphertext image data. This relationship and pattern are exploited to crack the ciphertext image.

In contrast, this paper adopts the SHA256 algorithm to compute the hash value of the plaintext image. The hash value is utilized to generate the bifurcation coefficients of the parameter-variabled coupled chaotic system. Consequently, even slight changes to the plaintext image lead to a complete alteration in the pseudo-random sequence generated by the system, causing a significant change in the resulting ciphertext image. As a result, the attacker cannot establish any meaningful connection between the plaintext image and the ciphertext image through minor modifications to the plaintext image. Therefore, the encryption algorithm presented in this paper effectively withstands differential attacks.

**Figure 10:**
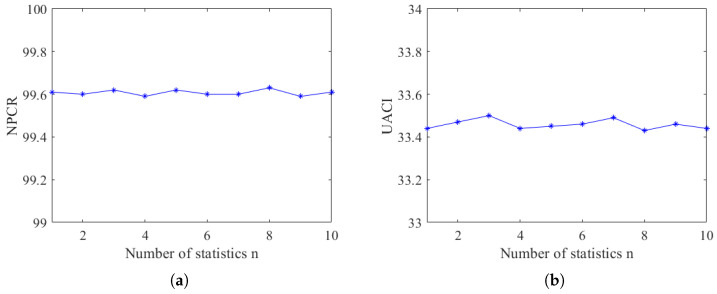
NPCR and UACI values. (**a**) NPCR values. (**b**) UACI values.

The analysis of anti-differential attacks typically relies on two parameters: NPCR (Number of Pixels Changing Ratio) and UACI (Unified Average Changing Intensity). Ideally, these parameters should have values of 99.6094% and 33.4635%, respectively [48], as expressed in Equations (32) and (33).

(32) $$\begin{array}{c} NPCR=\frac{1}{M\times N}\sum_{i=1}^{M}\sum_{j=1}^{N}E(i,j)\times 100\%\\ E\left(i,j\right)=\left\{\begin{array}{c} 0,D\left(i,j\right)=D^{\prime }\left(i,j\right)\\ 1,D\left(i,j\right)\neq D^{\prime }\left(i,j\right) \end{array}\right. \end{array}$$

(33) $$\begin{array}{c} UACI=\frac{1}{M\times N}\sum_{i=1}^{M}\sum_{j=1}^{N}\frac{|D\left(i,j\right)-D^{\prime }\left(i,j\right)|}{255}\times 100\%, \end{array}$$

where *D* and $D^{\prime }$ are the ciphertext pixel matrix after ciphertext encryption for specific differences, respectively. Using the Cameraman grayscale image as an example, ten sets of experiments were conducted for comparison, and each experiment changed one pixel point in the original Cameraman image to observe the changes in the NPCR and UACI values of the ciphertext image, as shown in Figure 10.

The results of this paper’s anti-differential performance analysis are compared with other literature and are shown in Table 6.

Table 6 demonstrates that when the hash value is not involved in the encryption process, the relative error of the performance index against differential attacks significantly deviates from the ideal value. However, the NPCR and UACI values of the final encryption scheme presented in this paper closely approach the ideal values. The relative error between NPCR and the ideal value is only 0.007%, and the relative error between UACI and the ideal value is 0.012%. These results indicate that the encryption algorithm exhibits strong performance against differential attacks.

### 6.6. Histogram Analysis

**Figure 11:**
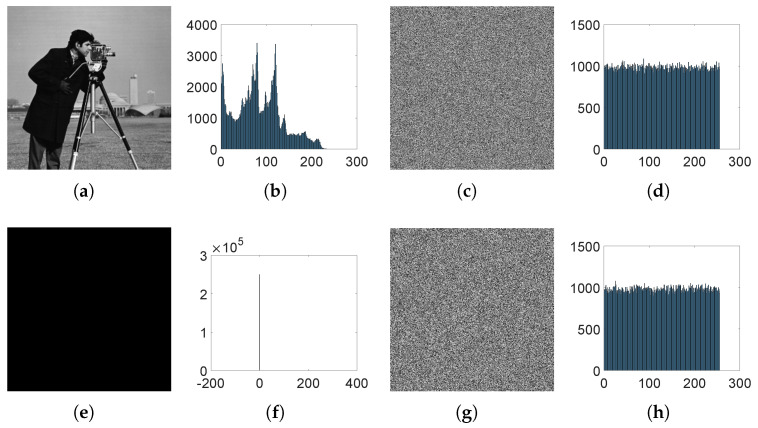
(**a**) Cameraman, (**b**) Pixel distribution histogram of Cameraman, (**c**) Cameraman’s ciphertext image, (**d**) Encrypted pixel distribution histogram, (**e**) Black, (**f**) Pixel distribution histogram of Black, (**g**) Black’s ciphertext image, (**h**) Encrypted pixel distribution histogram.

The image histogram describes the distribution of pixel values in an image. The pixel distribution of typical color images is usually uneven. If after encryption the original pixel distribution is disrupted and appears uniform, it is indicaive of the feasibility of the encryption algorithm. It can be observed from Figure 11 that the encryption algorithm in this paper can generate a ciphertext image with a uniform pixel distribution.

### 6.7. Correlation Test

The correlation coefficient serves as a numerical measure to assess the statistical relationship between two variables. In the original image, a strong correlation existed between connected pixels. If the correlation between connected pixels in the ciphertext image remained high, it often resulted in information leakage around the pixel, providing an opportunity for attackers to exploit statistical vulnerabilities. A robust encryption algorithm therefore must disrupt the correlation between pixels.

To achieve this, the following analysis focuses on the image in the vertical, horizontal, diagonal, and antidiagonal directions, employing Equations (34), (35), (36), (37), respectively.

(34) $$E\left(u\right)=\frac{1}{N}\sum_{i=1}^{N}u_{i},$$

(35) $$D\left(u\right)=\frac{1}{N}\sum_{i=1}^{N}{\left(u_{i}-E\left(u\right)\right)}^{2},$$

(36) $$\mathrm{cov}\left(u,v\right)=\frac{1}{N}\sum_{i=1}^{N}\left(u_{i}-E\left(u\right)\right)\left(v_{i}-E\left(v\right)\right),$$

(37) $$r_{uv}=\frac{\mathrm{cov}(u,v)}{\sqrt{D(u)}\sqrt{D(v)}},$$

where *u* and *v* represent the pixel values of adjacent pixels and $r_{uv}$ is the correlation of adjacent pixels.

**Figure 12:**
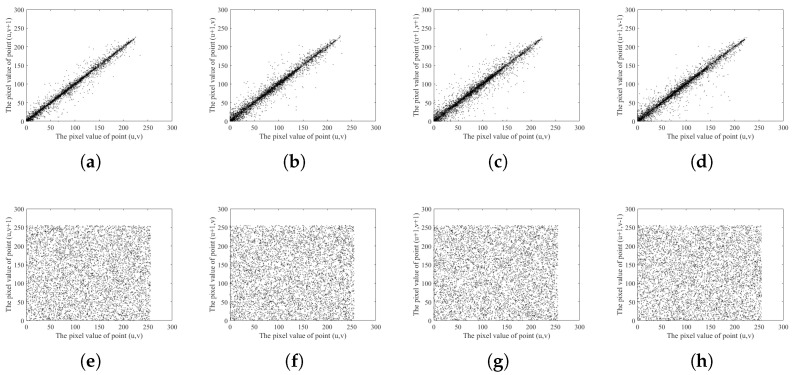
(**a**) Cameraman plaintext vertical pixel distribution; (**b**) Cameraman plaintext horizontal direction pixel distribution; (**c**) Cameraman plaintext diagonal direction pixel distribution; (**d**) Cameraman plaintext anti-diagonal direction pixel distribution; (**e**) Cameraman ciphertext vertical direction pixel distribution; (**f**) Cameraman ciphertext horizontal direction pixel distribution; (**g**) Cameraman ciphertext diagonal pixel distribution; (**h**) Cameraman ciphertext diagonal pixel distribution.

In this paper, according to the number of pixels, we decide to take 6000 sets of adjacent pixel values. The distribution of adjacent pixel values of the plaintext image and ciphertext image is shown in Figure 12. From Figure 12, we can see that the correlation of the ciphertext image is far lower than that of the plaintext image and close to zero; this shows that the pixels of the ciphertext images have almost no correlation, and the randomness is very strong, so the attacker cannot obtain any effective information from the ciphertext images.

### 6.8. Occlusion and Noise Attack Experiment

**Figure 13:**
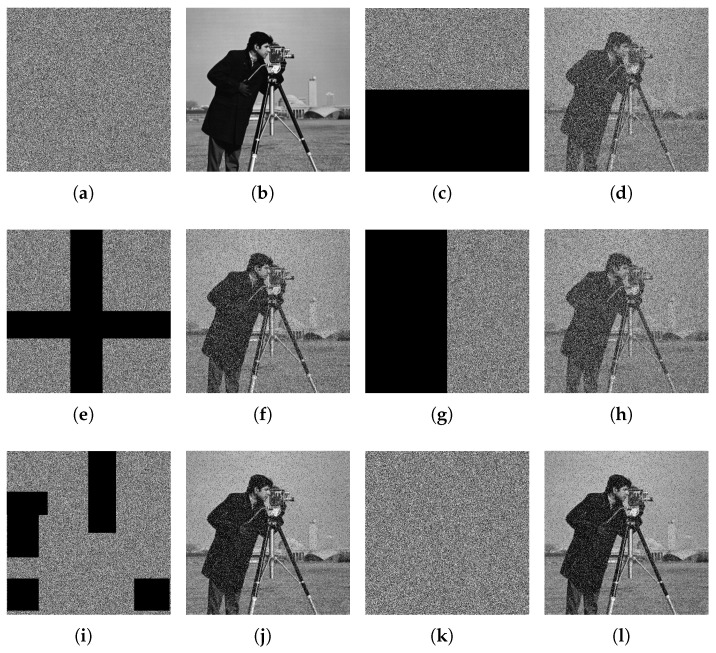

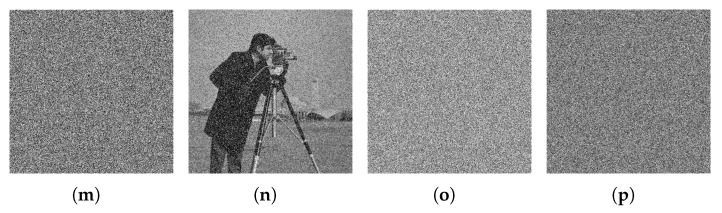
Occlusion and noise attack experiments. (**a**) Original ciphertext image; (**b**) Decryption image of (**a**); (**c**–**j**) Occlusion attack and corresponding decryption results; (**k**) Added salt-and-pepper noise with mean 0.1; (**l**) Decrypted image of (**k**); (**m**) Added salt-and-pepper noise with mean 0.2; (**n**) Decrypted image of (**m**); (**o**) Added Gaussian noise with a mean of 0.1 and a variance of 0.1; (**p**) Decrypted image of (**o**).

In the process of image transmission, the information is inevitably interfered with, and the algorithm with good performance should be able to recover the part of the information contained in the ciphertext image even if the part of the information is interfered with. In this section, we select occlusion attack, salt-and-pepper noise attack, and Gaussian noise attack to interfere with ciphertext images, and the test results are shown in Figure 13.

Our algorithm demonstrates strong resistance to occlusion attacks (Figure 13c,e,g,i) and salt-and-pepper noise attacks (Figure 13k,m) because these attacks only affect part of the image’s pixels. The hash value in our encryption algorithm is embedded multiple times, allowing for a high extraction success rate under such attacks. However, the algorithm is not effective against Gaussian noise attacks (Figure 13o). The main reason is that the hiding algorithm used in this study is based on the spatial domain, and Gaussian noise uniformly disrupts all pixels of the image. This prevents the encryption algorithm from correctly extracting the hidden hash value, thus failing to retrieve the key and successfully decrypt the image. Therefore, future research could explore more robust, frequency domain-based hiding algorithms to replace the current hiding algorithm.

## 7. Discussion

In this paper, we conducted a comprehensive review of existing chaos-based image encryption algorithms, identifying notable shortcomings in both chaotic systems and encryption structures. To address these issues, we proposed a general model of a parameter-variabled coupled chaotic system (PVCCS). This model effectively couples the variables and parameters in one-dimensional chaotic maps, resulting in a more complex high-dimensional system. Specifically, we developed a three-dimensional parameter-variabled coupled chaotic system (3DPVCCS) using logistic mapping and a pseudo-random sequence generation algorithm based on 3DPVCCS. Our experimental analysis demonstrates that 3DPVCCS outperforms one-dimensional chaotic systems across various metrics, exhibiting strong hyperchaotic characteristics. Thus, PVCCS proves feasible for generating more complex high-dimensional chaotic systems and chaotic sequences with enhanced randomness by coupling one-dimensional chaotic systems.

Following this, we designed a novel image encryption algorithm based on 3DPVCCS, which comprises three main components: pixel scrambling, pixel diffusion, and hash hiding. The integration of the pixel chain diffusion model with DNA encoding and decoding operations enhances the encryption complexity. Notably, the hash value used for sub-key generation is embedded into the ciphertext through hash value hiding, addressing the issue of key reuse in encryption algorithms that rely on plaintext information and significantly improving the algorithm’s practicality. Additionally, the inclusion of the plaintext hash value in the encryption process effectively mitigates the risk of differential cryptanalysis. Security and performance tests reveal that the encryption algorithm delivers excellent results.

However, our proposed algorithm still has areas that warrant further improvement and research. For example, the hash hiding can be performed using more robust and space-efficient hiding algorithms, provided that they are lossless. Additionally, exploring frequency domain hash hiding algorithms could make the algorithm resistant to Gaussian noise attacks, which is one of the shortcomings of the current algorithm. Our future work will focus on exploring the hardware implementation of the coupling method and applying the theoretical concepts to practical scenarios. Additionally, we will conduct a more rigorous and comprehensive analysis of the properties of the parameter-variabled coupled chaotic system (PVCCS), including a detailed investigation into its chaotic behavior and potential applications. Moreover, the proposed encryption algorithm can be further studied for downstream tasks involving large-scale and frequent secure data exchange.

## Figures and Tables

**Table 1:** DNA coding rule $F_{i}$.

Base	$F_{0}$	$F_{1}$	$F_{2}$	$F_{3}$	$F_{4}$	$F_{5}$	$F_{6}$	$F_{7}$
A	00	00	01	01	10	10	11	11
T	11	11	10	10	01	01	00	00
G	10	01	00	11	00	11	01	10
C	01	10	11	00	11	00	10	01

**Table 2:** DNA operations $O_{J}$.

$O_{1}$	A	T	G	C
A	A	T	G	C
T	T	A	C	G
G	G	C	A	T
C	C	G	T	A
$O_{2}$	A	**T**	**G**	**C**
A	A	T	G	C
T	T	C	A	G
G	G	A	C	T
C	C	G	T	A
$O_{3}$	**A**	**T**	**G**	**C**
A	A	G	T	C
T	T	A	C	G
G	G	C	A	T
C	C	T	G	A

**Table 3:** Results of the NIST SP800-22 and TestU01 test suite.

Test Suite	Test Items	P	Pass Ratio	Result
NIST SP800-22	monobit test	0.40918858223701704	50/50	Success
	frequency within block test	0.2068273229640186	50/50	Success
	runs test	0.4221833935716133	50/50	Success
	longest run ones in a block test	0.6052109259457924	50/50	Success
	binary matrix rank test	0.4239087697729736	50/50	Success
	dft test	0.6630425587345121	50/50	Success
	non overlapping template matching test	0.4299817488726386	50/50	Success
	overlapping template matching test	0.4914602095188029	50/50	Success
	maurers universal test	0.6190680234292043	50/50	Success
	linear complexity test	0.5589134031355517	50/50	Success
	serial test	0.57129332899815247	50/50	Success
	approximate entropy test	0.48940427208059406	50/50	Success
	cumulative sums test	0.50694543437526995	50/50	Success
	random excursion test	0.51035699243095794	50/50	Success
	random excursion variant test	0.530462378275446	50/50	Success
TestU01	SmallCrush	/	10/10	Success
	Crush	/	96/96	Success
	BigCrush	/	106/106	Success

**Table 4:** Comparison of keyspaces.

	Keyspace
This paper	$10^{237}$
Ref. [40]	$10^{34}$
Ref. [41]	$10^{51}$
Ref. [42]	$10^{60}$
Ref. [43]	$10^{60}$
Ref. [24]	$10^{70}$
Ref. [30]	$10^{75}$
Ref. [44]	$10^{77}$
Ref. [45]	$10^{120}$
Ref. [23]	$10^{126}$
Ref. [46]	$10^{128}$
Ref. [47]	$10^{158}$
Ref. [29]	$10^{200}$

**Table 5:** Information entropy analysis.

Images	Before Encryption	After Encryption
Lena	7.4474	7.9997
Cameraman	4.4925	7.9998
Black	0	7.9988
White	0	7.9988
Ref. [40]	7.4450	7.9994
Ref. [41]	7.4474	7.9994
Ref. [42]	7.2736	7.9994
Ref. [43]	7.4464	7.9925
Ref. [24]	7.4474	7.9923
Ref. [30]	7.4451	7.9974
Ref. [44]	7.4451	7.9975
Ref. [45]	7.4451	7.9994
Ref. [23]	7.4474	7.9874
Ref. [46]	7.4474	7.9912
Ref. [47]	7.4474	7.9972
Ref. [29]	7.4474	7.9973

**Table 6:** Anti-differential performance analysis.

	NPCR	UACI
Proposed	99.6217	33.4607
Ref. [40]	99.3684	33.6725
Ref. [41]	99.6113	33.4448
Ref. [42]	99.622	33.471
Ref. [43]	99.3684	33.6725
Ref. [24]	99.5921	33.3687
Ref. [30]	99.59	33.55
Ref. [44]	99.6091	33.4612
Ref. [45]	99.6082	33.4742
Ref. [23]	99.53	32.57
Ref. [46]	99.6077	33.4558
Ref. [47]	99.6025	33.4597
Ref. [29]	99.6208	33.4301

## Data Availability

All data generated or analyzed during this study are included in this published article.

## Abbreviations

The following abbreviations are used in this manuscript:

PVCCS	Parameter-Variabled Coupled Chaotic System
PSGM	Pseudo-Random Sequence Generation Method
3DPVCCS	Three-Dimensional Parameter-Variabled Coupled Chaotic System
